# Fogarty-assisted transcatheter embolization of a large renal high-flow arteriovenous fistula

**DOI:** 10.1186/s42155-022-00295-1

**Published:** 2022-04-07

**Authors:** Lena S. Becker, Jan B. Hinrichs

**Affiliations:** Institute of Diagnostic and Interventional Radiology, Medical School Hanover, Carl-Neuberg-Str. 1, 30625 Hannover, Germany

**Keywords:** Renal arteriovenous fistula, Balloon-assisted fistula embolization, Interventional radiology

## Abstract

**Background:**

Renal high-flow arteriovenous fistulas and its complications may involve high output heart failure, hematuria, hypertension or lethal hemorrhage.

**Case presentation:**

This case report covers the case of a 65-year-old male patient with a large renal high-flow arteriovenous fistula of the right kidney (RAVF), treated with balloon-assisted coil and liquid (n-Butyl Cyanoacrylate) embolization. By use of ballon-occlusion with an over-the-wire Fogarty catheter and advancement of a microcatheter through the lumen distal to the balloon during the transcatheter embolization of a high-flow RAVF, control of arterial blood flow is feasible by temporary occlusion of the afferent artery. This technique of flow modulation facilitates controlled deployment of embolization materials and decreases the risk of inadvertent distal embolization by use of only one 6-French (F) arterial sheath.

**Conclusions:**

Balloon-assisted embolization using a Fogarty occlusion catheter represents a feasible, safe and effective treatment option for the treatment of large, high-flow arteriovenous fistulas of the kidney.

## Introduction

Renal arteriovenous fistulas (RAVF) are a rare, anomalous connection between the renal artery and vein, without intervening capillary network and considerable clinical impact, potentially leading to hematuria, hypertension, local thrombosis, renal failure, high output heart failure or in case of rupture, severe hemorrhage with life-threatening bleeding (Carrafiello et al. 2011; Nagpal et al. 2016). Classified into congenital (25%), idiopathic (3–5%), and acquired causes (70%), recent developments of percutaneous interventions have increased the incidence of acquired RAVFs considerably (Duc 2021; Kato et al. 2006). Transcatheter embolization represents a potentially kidney-preserving, minimally invasive therapy option. However, size dimension, location as well as (high-) flow conditions pose substantial challenges concerning complete occlusion of the fistula without inadvertent embolization to other organs.

This report concerns the case of a 65-year-old male patient with decreasing renal function, presence of excessive proteinuria due to a high-flow AVF of the right kidney, treated by balloon-occlusion assisted embolization.

## Case report

**Fig. 1:**
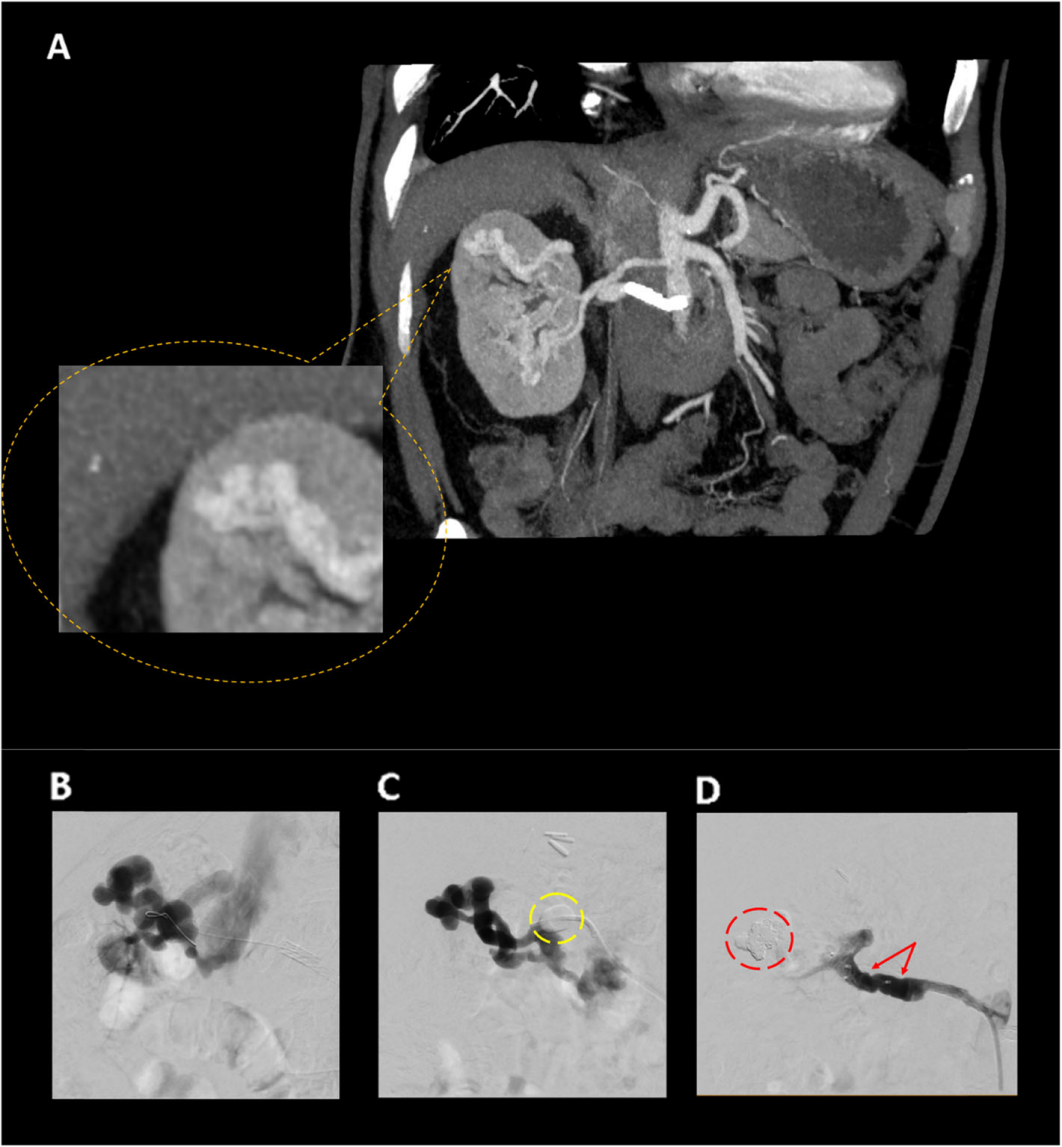
Initial arteriogram (**A**) of the right kidney with double artery perfusion, of which the caudal renal artery was stented in 1997. Presence of a subcapsular arteriovenous fistula in the upper renal lobe proved progressively increasing in size, with increasing risk of rupture, prompting indication for transarterial embolization. Catheterization of the high-flow RAVF showed immediate drainage into the inferior vena cava (**B**). Usage of a Fogarty occlusion catheter (**C**: yellow perforated circle) allowed for flow modulation and trans-catheter embolization via a mixture of coils and glue. Post-embolization angiogram (**D**) reveals complete occlusion of the AVF with coils (**D**: red perforated circle) and vascular plugs (**D**: red arrows)

A 65-year-old male patient was admitted to a University medical center for therapy of his progressively decreasing renal function, increasing proteinuria and enlarging arteriovenous fistula of the right kidney, first described in 2011 in a computed tomography (CT) scan. Patient history included chronic renal insuffiency and bilateral stenting of stenotic renal arteries (1997). Additional diagnoses of secondary hyperparathyroidsm, diabetes type 2, dyslipidemia, and arterial hypertension with recurring episodes of epistaxis were documented. Medication included five antihypertensives (alpha- and betablocker, angiotensin converting enzyme (ACE) antagonist, diuretic, calcium channel blocker), a sodium glucose co-transporter 2 blocker, and a statin. CT angiography (CTA) revealed double renal arteries in both kidneys, of which the more distal one of each side had been stented in 1997 using bare metal stents. No significant in-stent-stenosis could be detected at time of CTA or intervention (Fig. 1). The patient had been experiencing decreasing renal function with creatinin values of 227 umol/L and a progressively enlarging arteriovenous fistula of the stented right renal artery. Thus, increasing shunt flow was suspected responsible for renal impairment and indication for AVF occlusion was posed. The procedure was performed following an institutional standard operating procedure on a monoplane, ceiling-mounted angiographic system (Artis Q, Siemens Healthcare, Forchheim, Germany) under local anesthesia. The right femoral artery was accessed via a 6F vascular sheath (45 cm Destination® peripheral guiding sheath, Terumo Europe, Leuven, Belgium). An initial arteriogram of the upper right renal artery showed parenchymal enhancement of parts of the right kidney without signs of renal artery stenosis or AVFs. The subsequent arteriogram of the stented lower renal artery revealed the enlarged tortuous arteriovenous fistula (Fig. 1), though with restricted view of anatomic details due to the high flow in this vessel. Thereafter, a suitable diagnostic catheter was advanced through the stent into the artery and by using a 035” Rosen wire (Rosen curved wire guide, Cook Medical, Bloomington, United States of America (USA)), the sheath was advanced into the renal artery beyond the stent. For flow modulation and to achieve a stable and safe embolization position, an over-the-wire, compliant 5.5 F Fogarty occlusion catheter (Fogarty®, Edwards Lifesciences, Irvine, CA, USA) was advanced throughout the sheath into the renal artery and the occlusion balloon was inflated to the approximate size of the vessel (10 mm). The subsequent angiogram acquired through the lumen of the Fogarty catheter revealed a clear view of the afferent artery and draining vein with no parenchymal enhancement (Fig. 1). The afferent artery was probed with a microcatheter (Merit Maestro with Tenor 0.014 guidewire, Merit Medical Systems, Utah, USA) through the lumen of the Fogarty catheter as distally as possible. Detachable 3D coils (Concerto, Medtronic, Heerlen, The Netherlands) were used for framing (10 mm) and afterwards, pushable coils (VortX, Boston Scientific, Marlborough. MA, USA) for filling (4-6 mm). For complete occlusion, additional liquid embolization through the microcatheter was performed via off-label use of Histoacryl® (Histoacryl® n-Butyl Cyanoacrylate, Braun, Rubi, Spain). The microcatheter was removed and after precipitation of the glue, the occlusion balloon was deflated. During fluoroscopy, no embolization through the effernt vein was seen. Finally, two vascular plugs (AmplatzerPlug2 14 mm, Abbott Medical, Plymouth, MN, USA) were deployed through the 6 F sheath. Arteriography following embolization demonstrated occlusion of the fistula. No inadvertent distal embolization in other organs occurred and an ultrasound examination three days later documented total occlusion of the AVF. The patient was discharged with subjective well-being and is under active surveillance.

## Discussion

Though overall rare, RAVFs may demonstrate considerable clinical impact in case of presence of gross hematuria, hypertension, cardiac and renal failure or severe hemorrhage due to rupture (Carrafiello et al. 2011; Nagpal et al. 2016). The majority of AVFs are acquired and occur as a result of renal interventions (e.g. renal biopsy, surgery), blunt or penetrating trauma, inflammation or malignancy, though congential and idiopathic AVFs exist (Duc 2021; Kato et al. 2006). Indications for treatment in this patient included the progressive size increase, decrease of kidney function as well as the symptoms of arterial hypertension. Traditionally, surgical approaches with open resection and ligation of the renal artery have been preferred for patients with fistula-associated alterations of the cardiovascular system (Kuklik et al. 2017; Saliou et al. 1998; Campbell et al. 2009; Trocciola et al. 2005). However, more recently the advantages of less invasive and more modern treatment options, enabling preservation of the kidney, general decrease of peri-operative morbidity and mortality with high efficacy, have led to a preference for transcatheter embolizations. Despite the aforementioned benefits, this procedure type carries some risks for complications. A previous study by Uchikawa et al. described migration of n-Butyl Cyanoacrylate into the venous system in the majority of their reported cases (Uchikawa et al. 2015), while Abdel-Aal et al. reported migration of a coil into the pulmonary artery, which prompted additional interventional procedures for retrieval (Abdel-Aal 2014). Thus, some sort of embolization protection seems reasonable. In our case, we performed angiography and embolization of a large high-flow arteriovenous fistula of the right kidney, which possesses a scientifically proven risk of inadvertent distal embolization and/or reflux. The Fogarty occlusion catheter represents a cost-effective option amongst the group of balloon catheters with low risk of trauma due to its compliant nature. Using a balloon-occlusion technique, which combined flow control by the balloon and embolization through the lumen of the catheter by advancement of a suitable microcatheter, this technique enables a clear view of the anatomy in high-flow arteries and a safe embolization even with liquid embolic agents without the need for additional arterial or venous punctures and catheter maneuvers.

## Conclusions

Balloon-assisted embolization using a Fogarty occlusion catheter represents a feasible, safe and effective approach for the treatment of large, high-flow arteriovenous fistulas of the kidney.

## Data Availability

N.A.

## Abbreviations

CT (A): Computed tomography (angiography)
F: French
(R)AVF: (Renal) arteriovenous fistula
